# An electrokinetic-biocementation study for clay stabilisation using carbonic anhydrase-producing bacteria

**DOI:** 10.1007/s11356-023-29817-7

**Published:** 2023-09-13

**Authors:** Wilson Mwandira, Maria Mavroulidou, Anjali Satheesh, Michael John Gunn, Christopher Gray, Diane Purchase, Jonathan Garelick

**Affiliations:** 1 Division of Civil and Building Services Engineering, London South Bank University, London, UK; 2 London South Bank University, London, UK; 3 Faculty of Science and Technology, Middlesex University, London, UK; 4 Network Rail-Eastern Region, One Stratford Place, Stratford City, London, E20 UK

**Keywords:** Ground improvement, Electrokinetics, Carbonic anhydrase, CO_2_ capture, Biocementation

## Abstract

This study investigates the feasibility of biocementing clay soil underneath a railway embankment of the UK rail network via carbonic anhydrase (CA) biocementation, implementing the treatments electrokinetically. Compared to previous biocementation studies using the ureolytic route, the CA pathway is attractive as CA-producing bacteria can sequester CO_2_ to produce biocement. Clay soil samples were treated electrokinetically using biostimulation and bioaugmentation conditions to induce biocementation. The effects of the treatment were assessed in terms of undrained shear strength using the cone penetration test, moisture content, and calcium carbonate content measurements. Scanning electron microscopy (SEM) analyses were also conducted on soil samples before and after treatment to evaluate the reaction products. The results showed that upon biostimulation, the undrained shear strength of the soil increased uniformly throughout the soil, from 17.6 kPa (in the natural untreated state) to 106.6 kPa. SEM micrographs also showed a clear change in the soil structure upon biostimulation. Unlike biostimulation, bioaugmentation did not have the same performance, although a high amount of CaCO_3_ precipitates was detected, and bacteria were observed to have entered the soil. The prospects are exciting, as it was shown that it is possible to achieve a considerable strength increase by the biostimulation of native bacteria capturing CO_2_ while improving the soil strength, thus having the potential to contribute both to the resilience of existing railway infrastructure and to climate change mitigation.

## Introduction

In the context of engineering sustainability, ground improvement is increasingly used for sites which contain unsuitable soil for construction or for the remediation of existing infrastructure earthworks. Traditional methods of chemical ground improvement include the addition of small percentages of Portland cement (Yoobanpot et al. 2017) or lime (Ghorbani et al. 2019; Kichou et al. 2022; Zhang et al. 2015a, b) and also fly ash (Abdullah et al. 2020; Deepak et al. 2021). Recent options include more innovative cements such as alkali-activated cements/geopolymers (Ghadir and Ranjbar 2018, Mavroulidou et al. 2022, 2023) or biocements, produced by the metabolic actions of microorganisms through various processes, including ureolysis (Omoregie et al. 2021; Phillips et al. 2013), photosynthesis (Irfan et al. 2019), denitrification (Jain et al. 2021; O’Donnell et al. 2017), and methane oxidation (Ganendra et al. 2014). This paper proposes instead the carbonic anhydrase metabolic pathway, as an innovative eco-friendly and sustainable soil stabilisation method, which can consume captured waste CO_2_ or CO_2_ from the atmosphere to precipitate CaCO_3_ which binds together (biocements) soil particles. In this paper, carbonic anhydrase is produced by *Bacillus licheniformis*, an indigenous bacterium, isolated from the project site and screened for biocementation. The CA biocementation pathway involves a multi-step chemical reaction process: CA utilises CO_2_ forming hydrated aqueous CO_2_ (aq) (Eq. 1), which reacts with water to form H_2_CO_3_ (Eq. 2).

1 $${\mathrm{CO}}_{2}\left(\mathrm{g}\right)\leftrightarrow {\mathrm{CO}}_{2}\left(\mathrm{aq}\right)$$

2 $${\mathrm{CO}}_{2}\left(\mathrm{aq}\right)+{\mathrm{H}}_{2}\mathrm{O}\left(\mathrm{aq}\right)\leftrightarrow {\mathrm{H}}_{2}{\mathrm{CO}}_{3}\left(\mathrm{aq}\right)$$

These products further ionise to form CO_3_^2−^ and H_2_O (Eq. 3). To form a biocement via biomineral precipitates, the metal ion, e.g., Ca^2+^, reacts with CO_3_^2−^ (Eq. 5), forming calcite where the CA enzyme serves as nucleation sites (Kahani et al. 2020).

3 $${\mathrm{H}}_{2}{\mathrm{CO}}_{3}\left(\mathrm{aq}\right)\leftrightarrow {\mathrm{H}}^{+}+{\mathrm{HCO}}_{{3}}^{-}\left(\mathrm{aq}\right)$$

4 $${2\mathrm{HCO}}_{{3}}^{-}+{2\mathrm{OH}}^{-}\leftrightarrow {2\mathrm{CO}}_{{3}}^{2-}+{2\mathrm{H}}_{2}\mathrm{O}$$

5 $${\mathrm{Ca}}^{2+}\left(\mathrm{aq}\right)+{\mathrm{CO}}_{{3}}^{2-}\left(\mathrm{aq}\right)\leftrightarrow {\mathrm{CaCO}}_{3}\left(\mathrm{s}\right)\downarrow$$

This paper presents a feasibility study of using electrokinetics (EK) to implement CA biocementation treatments to stabilise problematic geomaterials of the UK railway network, addressing climate change adaptation (infrastructure resilience) and mitigation. The rationale behind using EK is that it has several advantages over the more traditional methods of delivering agents to the soil that will bring about cementation. In particular, introducing agents by a pressure-driven injection system in fine-grained soil encounters various problems related to the low permeability of the soil. The distance of penetration from the injection point may be low, and the use of high pressures to increase penetration can lead to ground heave and even fracturing of the ground and blow-out failures of the injection system. There may be clogging around the injection point. Attempting to inject CO_2_ gas directly into the soil can lead to large voids, altering the soil structure. In contrast, using EK can lead to more homogeneous biocementation of the soil as electrically charged chemicals (i.e. ions) are transported more effectively into the soil.

The proposed method is novel as it combines CO_2_ capture with biocementation to treat fine-grained soils, and to the authors’ knowledge, this is the first study to combine carbonic anhydrase biocementation with EK, as the very few studies that attempted to implement biocementation treatments for fine-grained soils electrokinetically all used the ureolytic biocementation pathway (Keykha et al. 2014, Safdar et al. 2020, 2021a, b).

## Materials and methods

### Soil sample characteristics

**Fig. 1:**
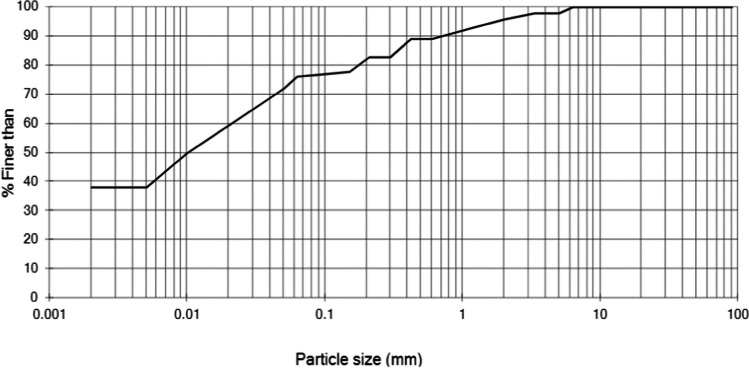
Particle size distribution of the soil used in this study

The soil used in this study was taken from a trial pit from the Prickwillow site of the East Anglia railway network. The soil used was found at 1.2–2.2-m depth and was characterised as sandy silty clay based on its particle size distribution (with a clay content of approximately 39%). Duplicate samples were tested to establish the physico-chemical characteristics of the soil. Figure 1 and Table 1 show the particle size distribution of the tested sample portion (from sieving followed by hydrometer testing according to BS 1377-2:1990) and other basic soil characteristics.

**Table 1:** Basic physico-chemical properties of sandy silty clay soil from Prickwillow, East Anglia, UK

Property	Value	Test/standard
Liquid limit: % w/w*	63	Cone penetrometer; BS 1377-2: 1990 (BSI 1990)
Plastic limit: % w/w	33	BS 1377-2: 1990 (BSI 1990)
Plasticity index: % w/w	30	BS 1377-2: 1990 (BSI 1990)
Organic matter content: % w/w	3.9	Loss of ignition: ASTM D2974-14 (ASTM 2014)
Natural gravimetric moisture content: % w/w	47.5	BS EN ISO 17892: Part 1: 2014
pH (of soil suspended in distilled water)	7.7	BS ISO 10390:2005 (BSI 2005)
Natural CaCO_3_ content (%)	4.82	Acid washing; ASTM D4373-21 (ASTM 2021)

### Microbiological study

#### Isolation and characterisation of CA-producing bacteria

To isolate bacteria, 1 g of soil sample was diluted in 10 mL of sterile deionised water. The thoroughly homogenised soil suspension in sterile water was diluted from 10- to 10,000-fold using sterile water and plated on a peptone agar medium spiked with 3 mM *p*-nitrophenyl acetate (p-NPA) (Sigma-Aldrich), used as an indicator for CA-positive-producing bacteria. Colonies of bacteria with the CA-producing ability presented an intense yellow colouration due to the hydrolysis of *p*-NPA into para-nitrophenol (pNP) (Sigma-Aldrich). The nutrient agar was incubated at 30 °C for 72 h to allow the growth of colonies identified in the plate media at the end of the incubation period according to the protocol followed in Sharma and Kumar (2021).

The identification of microbes was performed using the matrix-assisted laser desorption/ionization time-of-flight/time-of-flight tandem mass spectrometry (MALDI-TOF/TOF MS) proteomic-based biotyping approach (Singhal et al 2015), using a Bruker Daltonics MALDI Biotyper. Mass spectrometry is an analytical technique in which samples are ionised into charged molecules and their mass-to-charge ratios (m/z) can be measured. Microbial identification using MALDI-TOF MS is based on the principle that every microorganism has a specific protein composition, which gives its characteristic and unique mass spectra (MS). The peptide mass fingerprint (PMF) of unknown organisms is compared against those contained in the database by matching the masses of biomarkers of unknown organisms with the proteome database. Namely, in PMF matching, the MS spectrum of unknown microbial isolates is compared with the MS spectra of known microbial isolates in the database. A typical mass range m/z of 2–20 kDa was used for species-level identification of microbes (Benagli et al. 2012; Murray 2012).

#### Microbial growth and CA production and characterisation

The CA enzyme was determined colourimetrically to determine CA activity (Martin et al. 2009). The identified bacterial isolates that produced intense yellow colouration were further screened for CA enzyme activity in nutrient broth. The activity for p-nitrophenyl acetate hydrolysis was determined at room temperature in a reaction mixture (1.35 mL) containing freshly prepared 3 mM *p*-nitrophenyl acetate in phosphate buffer (0.13 M and pH 7.2). A pure colony was prepared aseptically in 50 mL nutrient broth for the CA enzyme activity assay and incubated at 37 °C for 24 h (37 °C was found to be the optimal temperature for CA enzyme activity—see Fig. 3c). The activity for p-NPA hydrolysis was determined using a method described by Armstrong et al. (1966). Namely, the reaction proceeded for 5 min, and the change in optical density (OD) readings at 348 nm were measured by UV-vis spectrophotometry (Jenway 6305, Bibby Scientific, Staffordshire, UK). Then the CA activity was characterised by the amount of *p*-nitrophenol produced per unit of time, and enzyme activity was expressed in terms of *U* according to Eq. 6.

6 $$\mathrm{CA}\;\mathrm{activity}\left(\frac U{\mathrm{mL}}\right)=\frac{\left(\Delta A_{348}T-\Delta A_{348}B\right)\times1000}{5\times\mathrm{Volume}}$$

where $\Delta {A}_{348}T$ is the final reading of absorbance; $\Delta {A}_{348}B$ is the initial uncatalysed reaction (blank) at a wavelength of 348 nm; and 1 U (μmol/min) is defined as the amount of the enzyme that catalyses the conversion of 1 μmol of substrate per minute. UV-vis spectrophotometry measured microbial growth, which recorded optical density (OD) readings at 600 nm for 96 h. Note that although all phases of bacteria growth can generally be seen within 24–30 h, this extended time period of 96 h was adopted to observe when the carbonic enzyme is released to establish the optimal secretion of carbonic anhydrase by the newly isolated strain. As temperature would affect the growth and reproduction of CA-producing bacteria and consequently the activity of enzymes, temperatures were set at 5 ℃, 15 ℃, 25 ℃, 30 ℃, and 37 ℃, respectively. After sterile inoculation, the medium was cultured in a constant temperature oscillation incubator (120 rpm) at the above-mentioned temperatures for 24 h. Then the OD_600_ value and carbonic anhydrase activity in the medium solution were measured as described previously. Based on this, three candidate strains were thus selected for further study (Mwandira et al. 2023). In this paper, experiments were conducted using one of these CA-producing isolates, i.e., *Bacillus licheniformis*. The strain *B. licheniformis* from the project site was cultured in a liquid medium of 5.0 g/L peptone, 1.50 g/L yeast extract, 1.50 g/L beef extract, and 5.0 g/L sodium chloride. Inoculant solutions were incubated aerobically at 37 °C and shaken at 120 rpm for approximately 48 h. A previously established relationship by the authors between bacterial plate counts and optical density at a wavelength of 600 nm (OD_600_) was used to determine a final *B. licheniformis* density of 9 × 10^8^ cells/L within the growth medium following incubation. This cell concentration was diluted in the applied bioaugmentation solution.

### Electrokinetic treatment schemes

**Fig. 2:**
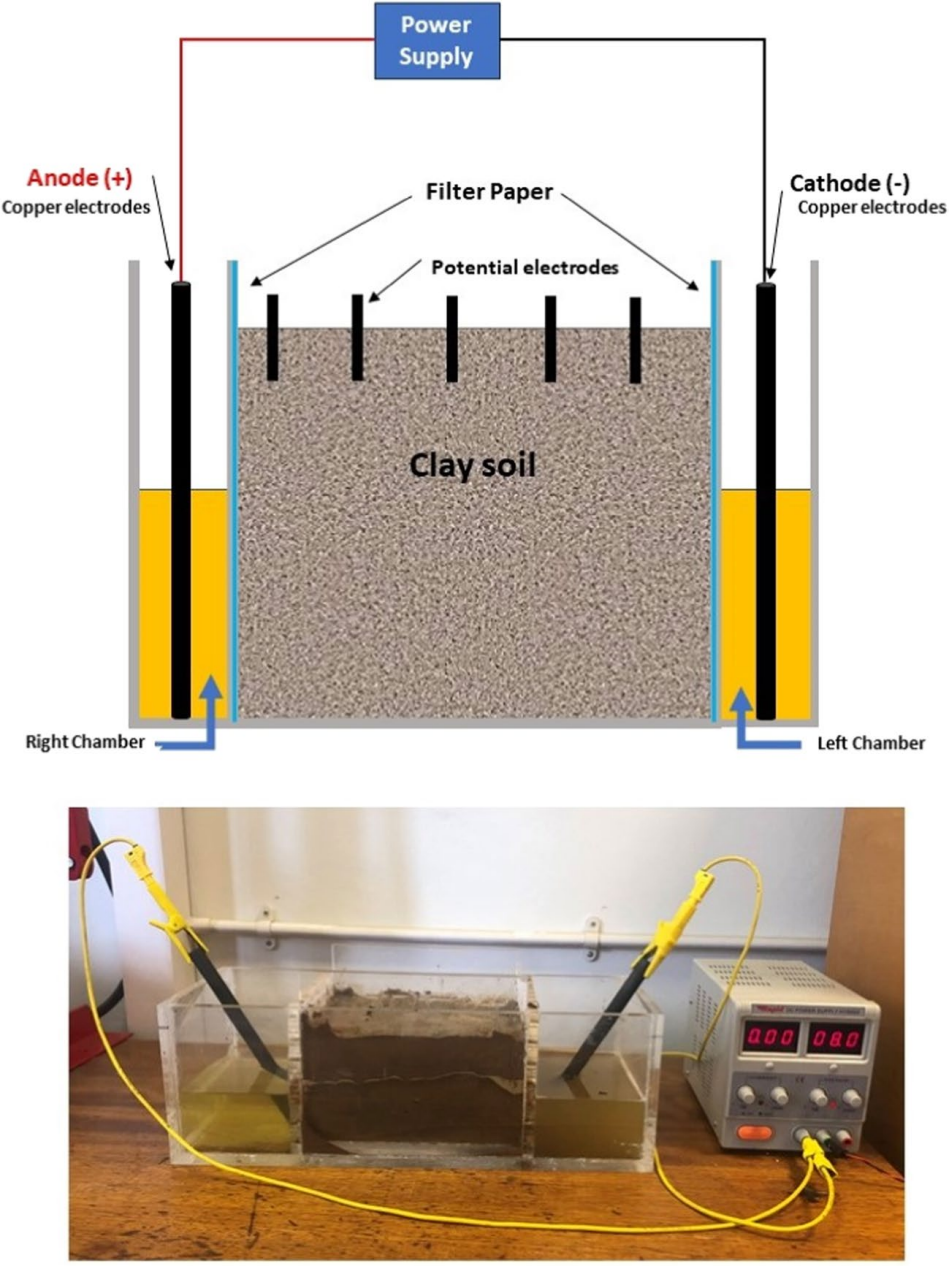
A schematic diagram and photograph of the EK treatment setup used in this study

The EK experiment was conducted in a laboratory environment at ambient temperature. A schematic diagram of the EK-biocementation test is shown in Fig. 2. The setup consisted of three chambers separated by perforated walls: anode chamber, soil compartment, and cathode chamber. The EK tank used had internal dimensions of 210 mm length × 160 mm width × 140 mm depth. To avoid material loss, filter papers were used at both ends of the specimen. The method adopted for EK biocementation was previously described by Safdar et al. (2020, 2021a, b) for treating organic soil. Once the filter papers were placed, the clay soil was statically compacted into the cell in three layers at its natural bulk density using a hydraulic frame. The soil sample was placed into the main compartment, and the two small compartments were used to supply different solution types: biostimulation, bioaugmentation, or cementation solutions. In this study, biostimulation involved modifying the environment to stimulate existing carbonic anhydrase bacteria capable of biocementation, whereas bioaugmentation involved the addition of pre-grown *B. licheniformis* (the carbonic-producing bacteria culture) to enhance microbial populations to improve biocementation. Table 2 shows the treatment solution constituents used in this study. Calcium acetate was used as a calcium source to avoid acid fronts and the destruction of bacterial cells in EK biocementation as recommended in Terzis et al. (2020). Namely, Terzis et al. compared the performance of calcium chloride against calcium acetate and calcium lactate as calcium sources in EK biocementation. They argued that microorganisms are vulnerable to hypochlorous acid (formed due to the production of active chlorine as Cl^−^ anions undergo oxidative reactions at the anode when CaCl_2_ is used); hence, they recommended calcium acetate and calcium lactate as calcium sources for EK-biocementation.

**Table 2:** Treatment solution constituents

Constituent	Solution type		
	Biostimulation	Nutrients	Cementation solution
Yeast extract	10 g/L	1.5	-
Peptone	-	5.0	-
Beef extract	-	1.50	-
Sodium chloride	-	5.0	-
Calcium acetate	-	-	0.1 M
Sodium bicarbonate	100 mM	-	0.1 M
Zinc sulphate	1 µM	-	-

As shown in Table 3, the EK biocementation treatments using nutrient broth solution and the cementing reagents-only tests were supplied all in one solution divided equally in the two electrolyte compartments. In the bioaugmentation tests, the 24-h pre-cultured *B. licheniformis* bacteria at a concentration of 1 × 10^8^ cfu/mL were introduced into the two chambers first (1–3 days) and the cementing reagents later (4–14 days). The intention was to move bacteria from the chambers into the soil electrokinetically (rather than the reaction products only), to verify this, qualitative plating of the soil before and after treatment was performed (see “Variation of temperature, pH, and electrical conductivity during EK-biocementation”). In the biostimulation case, the biostimulation solution was implemented first (1–3 days) and the cementing reagents later (4–14 days). During the EK treatment, polarity was reversed every 24 h, as recommended in the literature, to maintain pH in a particular range for better uniformity, hence the effectiveness of the treatment (Ciblak et al. 2012, Keykha et al. 2014), but also to prevent high-pH gradients that could harm the bacteria (Mena et al. 2016; Safdar et al. 2020, 2021a, b). During the experiments, temperature, pH, and electrical conductivity (EC) were monitored in both electrolyte chambers. A constant voltage of 8 V was applied between the anode and cathode (spaced 20 cm apart), giving a gradient of 0.4 V/cm, which is considered safe for bacteria survival (Safdar et al. 2021a, b).

**Table 3:** Summary of tests in this study

S/no	Cases	Treatment days	Electrolyte volume	Solution type
1.	Nutrients only	1–14	3.2 L	Nutrients
2.	Cementation solution only	1–14	3.2 L	Cementation solution
3.	Bioaugmentation	1–3	3.2 L	Bioaugmentation with *B. licheniformis* at 1 × 10^8^ cfu/mL concentration
		4–14	3.2 L	Cementation solution
4.`	Biostimulation	1–3	3.2 L	Biostimulation
		4–14	3.2 L	Cementation solution

### Soil property measurements

After EK-biocementation, the following measurements were performed at the top, middle, and bottom of each specimen: cone penetration test, gravimetric water content (i.e., the ratio of the mass of water present to the oven-dry mass of the soil sample), and CaCO_3_ content determined using the acid-washing technique. In this technique, an oven-dried mass of the soil samples was measured before and after an acid wash in a 2-M solution of HCl. Natural CaCO_3_ was determined first for the untreated soil (see Table 1), so that any additional CaCO_3_ measured in the treated soil samples can be attributed to the applied treatment. The cone penetration tests were performed according to the procedure prescribed in BS 1377-2: 1990 (BSI 1990) for liquid limit determination using a standard laboratory cone penetration apparatus. Then, the relationship between the cone penetration and the undrained shear strength *S*_u_ (kPa) was determined by Eq. 7, according to Leroueil and Le Bihan (1996).

7 $${S}_{u}=\frac{9.8 \times K \times M}{{P}^{2}}$$

where *M* is the mass of the cone (i.e., 80 g for the standard BS cone used), *P* is the cone penetration in mm, and *K* is a parameter depending on the angle of the cone. For an apex angle of 30°, which is the angle of the British Standard penetration cone, values of 0.8 or 0.85 were suggested for remoulded clays (as cited in Leroueil and Le Bihan 1996); a *K* value of 0.85 was therefore used here.

Additionally, the microstructure of the biominerals formed was analysed using scanning electron microscopy (SEM) with a Thermo Scientific Pharos FEG-SEM (Thermo Fisher Scientific, USA) of high vacuum mode and 15 kV acceleration voltage and a backscattered detector.

## Results and discussion

### Microbiological analysis results

The newly isolated carbonic anhydrase bacteria identified as *Bacillus licheniformis* were used for bioaugmentation. As stated in Safdar et al. (2022), the American Type Culture Collection (ATCC) classifies the isolated strain as of Biosafety Level (BSL) 1 (i.e. not known to consistently cause disease in healthy adults, and of minimal potential hazard to laboratory workers and the environment), and similarly, the BacDive database for standardised bacterial information classifies the strain into Risk group 1 (biological agents which are unlikely to cause disease in an individual) according to the German Federal Institute for Occupational Safety and Health. *Bacillus licheniformis* is a well-known carbonic-anhydrase-producing bacterium (Han et al. 2018; Zhao et al. 2019).

**Fig. 3:**
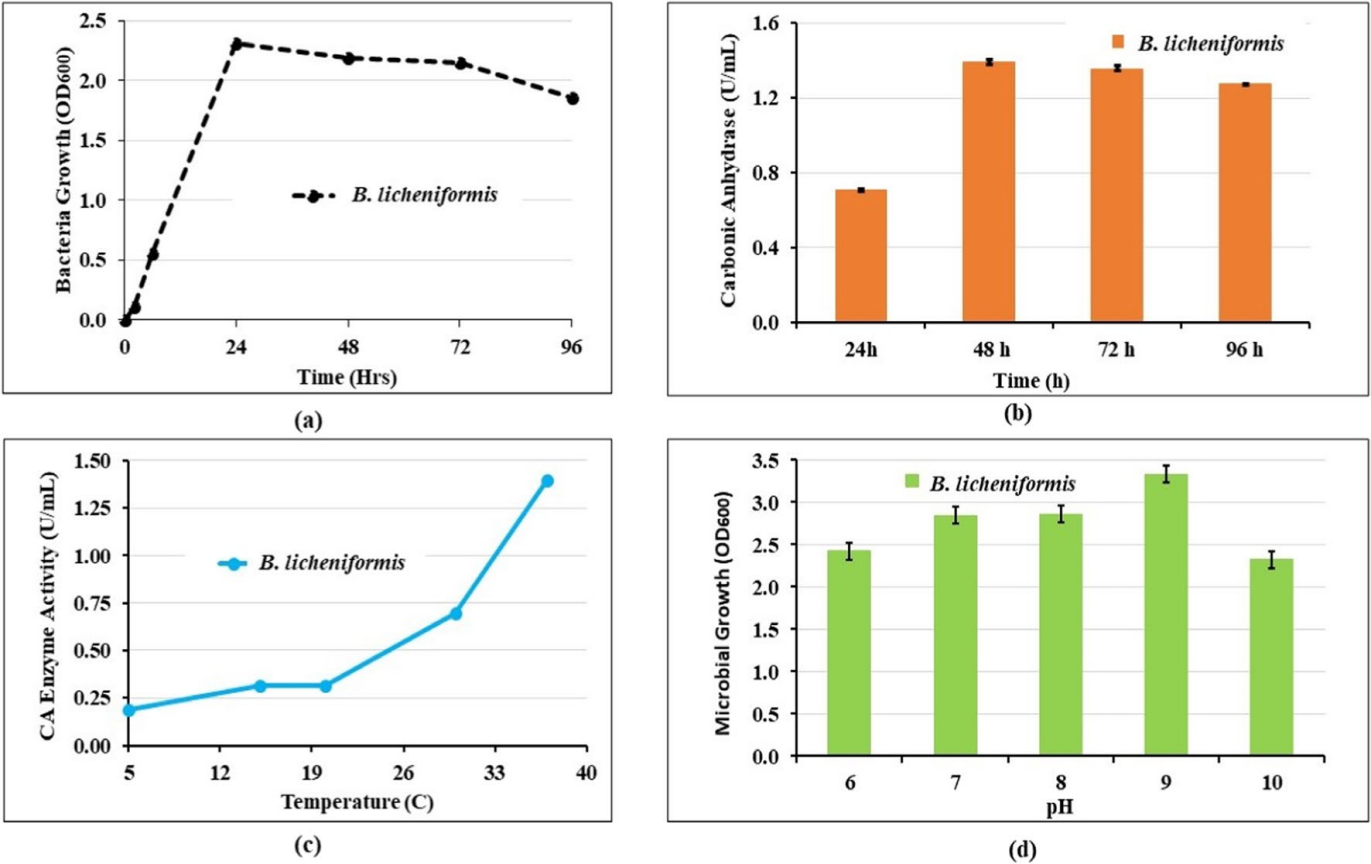
Optimal growth characteristics of *B. licheniformis* isolated from the site used for bioaugmentation case: **a** microbial growth; **b** carbonic anhydrase (CA) enzyme production; **c** temperature; **d** pH

The optimal growth characteristics of *B. licheniformis* are shown in Fig. 3. The interest was to see when the bacteria would grow most. *B. licheniformis* reached the maximum growth at 24 h with optimum growth pH of 9 and temperature of 37 ℃. OD_600_ measurements are typically used to determine the growth stage of a bacterial culture as a low-cost and effective method to measure bacterial growth (Beal et al. 2020; Zhang et al. 2015a, b). OD_600_ measurements help ensure that cells are harvested at an optimum point corresponding to an appropriate density of live cells. OD_600_ does not differentiate between bacteria and other particles. The shape of the growth phases is highly dependent on the physiological state of the inoculum. As the initial inoculum was harvested from a culture in the early stationary phase, the cells were still metabolically active and thus, the lag phase was relatively short, and the cells were able to enter the log phase in a shorter period of time (as seen in the growth curve). The growth rate (log phase slope) is commensurate with what we observed previously. At the stationary phase, if the medium contains a high background of dead cells, this could result in misleading results.

The quantitative analysis of the CA enzyme activity (Fig. 3c) showed that *B. licheniformis* had higher specific CA activity values of 2.31 U/mL in comparison to previously isolated CA-producing bacteria: *B. schlegelii* (0.0453 U/mL) (Muley et al. 2014) and *B. altitudinis* (0.695 U/mL) (Jaya et al. 2019). The newly isolated bacterium thus showed good potential for CA biocementation.

### Electrokinetic treatment results

#### Variation of temperature, pH, and electrical conductivity during EK-biocementation

**Fig. 4:**
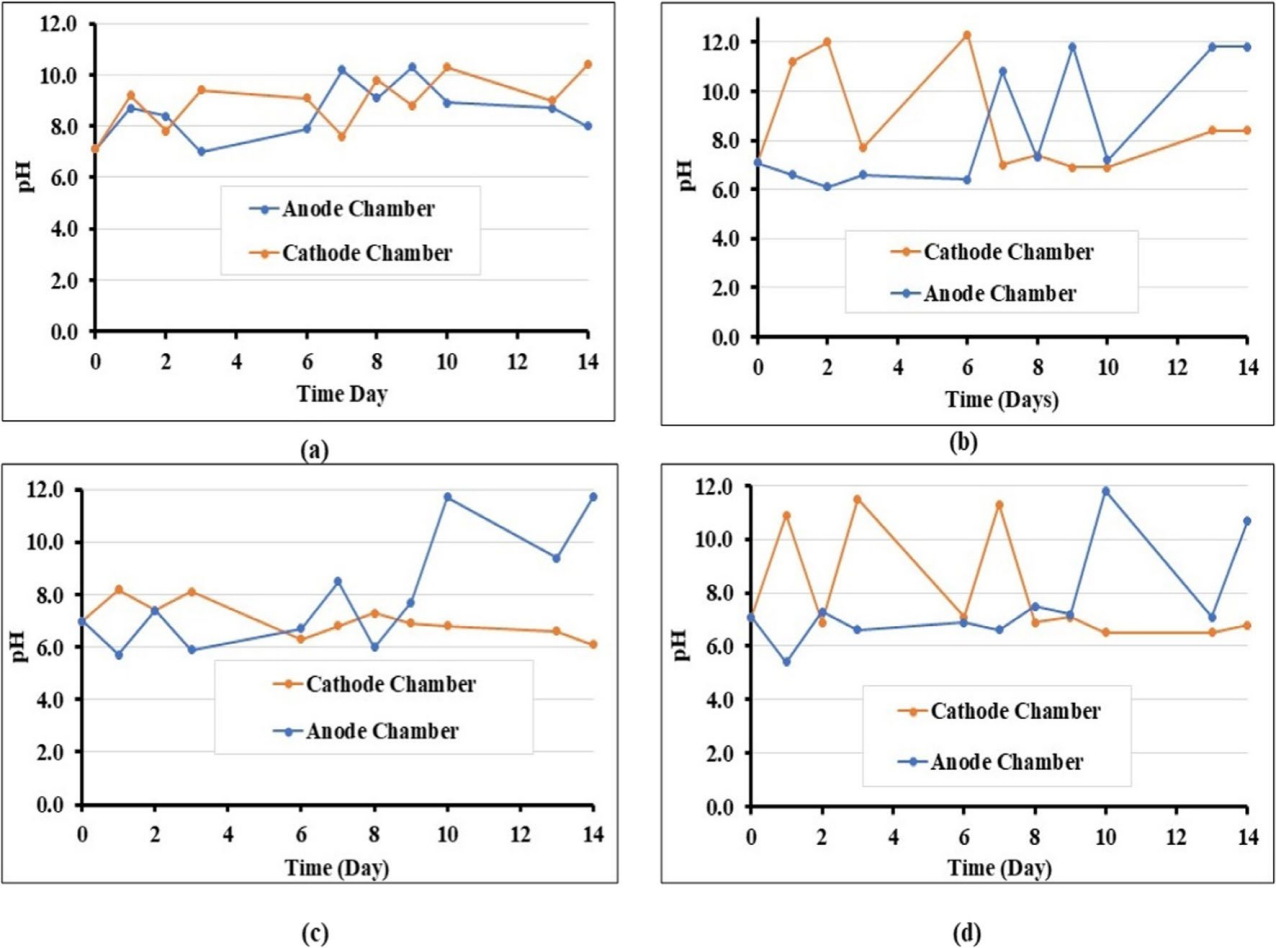
pH changes of anode and cathode chambers during various treatment procedures: **a** nutrients only; **b** cementation solution only; **c** biostimulation; **d** bioaugmentation using *B. licheniformis*. Note: “Anode” and “Cathode” chambers refer to the electrolyte chamber function when injection started

Temperature changes were generally uniform and followed the changes in room temperature. The pH values of electrolytes in the anode and cathode chambers were monitored for changes because pH is a critical factor affecting CaCO_3_ precipitation (Keykha et al. 2017). The pH in both electrolyte chambers (original anode and cathode chambers) at different times is shown in Fig. 4a, b, c, and d for nutrients only, cementation only, biostimulation and bioaugmentation, respectively. For all cases, the initial pH was around 7.0; subsequently, the pH frequently rose and decreased. As it is well known, in EK, large pH gradients are caused by the transport of protons and hydroxyl ions generated by water electrolysis on the electrode surfaces (Barba et al. 2017). These changes are due to hydrolysis upon electric current application, producing oxygen and hydrogen; water oxidation at the anode generates an acidic medium, whereas the reduction at the cathode produces a basic medium that causes a pH gradient between the two electrodes. Electrokinetic phenomena then cause constant variation in pH at any point, triggered by the motion of charged particles, through the electrolyte, due to the potential field applied; pH fluctuations will also occur as chemical species react with each other, thus affecting local electric charges. In this work, polarity reversal was applied to control the pH, avoiding conditions which are not conducive to calcite precipitation and biocementation. Namely, an alkaline environment is required for calcite precipitation to occur during biocementation (Omoregie et al. 2021; Seifan et al. 2016). In contrast, a pH of less than 5 would lead to calcite dissolution. pH control was also required to avoid large pH gradients, which could be unfavourable for bacteria (Mena et al. 2016). The polarity reversal explains the zigzag pattern of pH changes shown in Fig. 4 as anode and cathode chambers alternate functions. The pH variations with polarity reversal at the anode and cathode have a similar trend, as reported previously (Azhar et al. 2018). These changes in pH upon reversal of polarity are however buffered due to the composition of the different electrolytes (as shown in Table 2); for example, calcium acetate is a soft base which acts as a pH buffer to balance the production of acids. Previously reported pH values of EK of purely distilled water showed that final pH values could be pH 5.8 and pH 10.7 at the anode and cathode, respectively (Ciblak et al. 2012). In our study, the solutions raised the pH compared to when purely distilled water was used; namely, the pH ranged from 6.2 to around pH 11.80. In the nutrient-only case, the initial pH was 7.10 in both chambers containing the solutions (anode and cathode chambers), which subsequently increased to 8.70 and 9.20, respectively. This increase and decrease in electrolyte pH could be attributed to anions and cations moving from the electrode chamber into the soil chamber and back into the chambers. This phenomenon causes the pH in both chambers to increase and decrease depending on the present ions in the soil (Gidudu and Chirwa 2022; Mena et al. 2016). Compared to other cases investigated, the nutrients only did not have drastic zigzag changes due to the limited amounts of ions involved compared to bioaugmentation, biostimulation, and cementing solution which contained calcium acetate. Conversely, due to its composition, the cementation-only solution had high quantities of anions and cations compared to nutrients only, and for this reason, pH changes were observed to fluctuate from pH 7.1 initially to pH 6.60 and pH 11.20 at the anolyte and catholyte, respectively. Overall, the pH changes could reflect the chemical reactions occurring during biocementation.

**Fig. 5:**
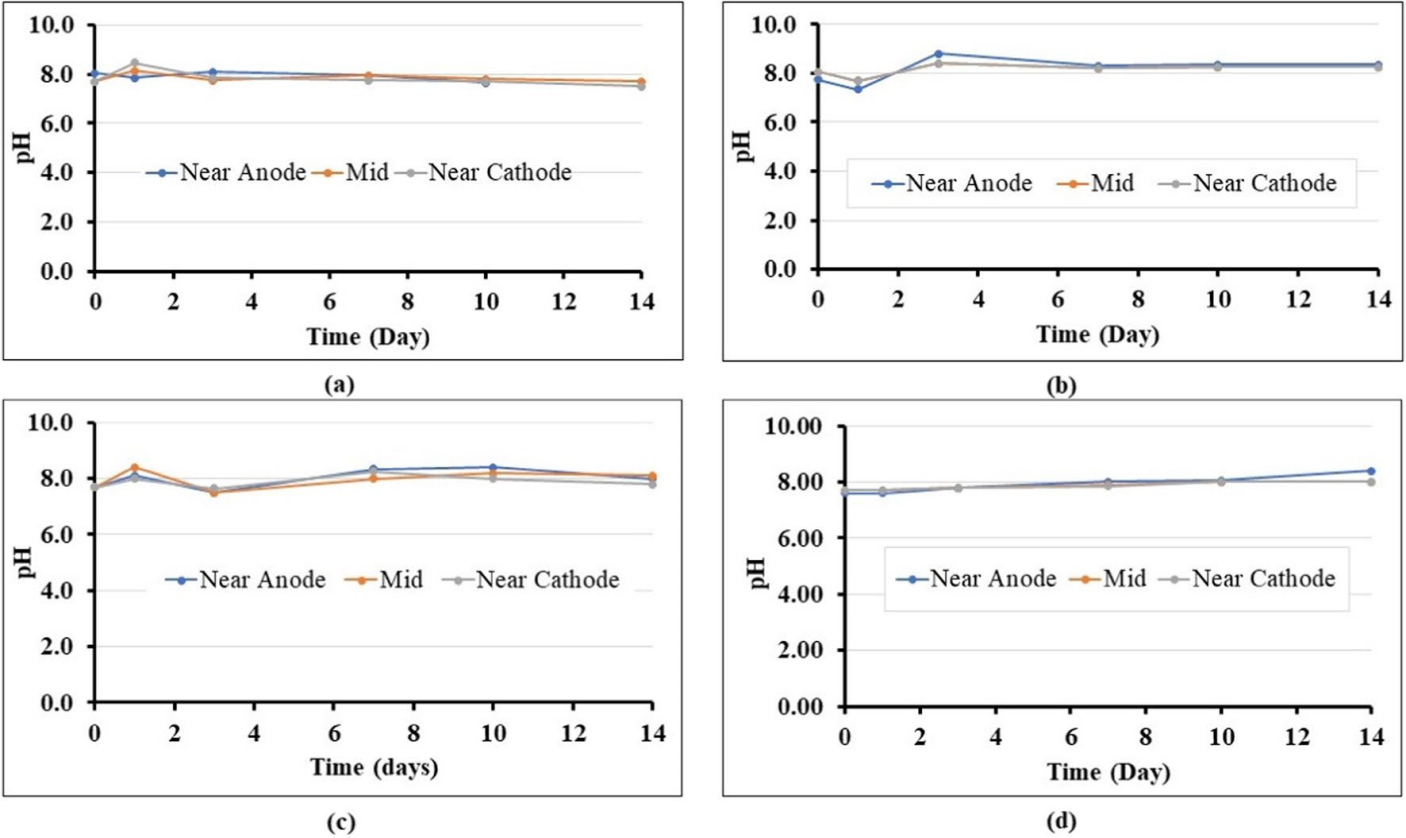
pH changes in soil chamber during EK treatments, next to the electrolyte chambers and in the mid-point of soil sample: **a** nutrients only; **b** cementation solution only; **c** biostimulation; **d** bioaugmentation using *B. licheniformis*. Note: “Anode” and “Cathode” refer to the electrolyte chamber function when injection started

The results of pH changes in the electrolyte chambers were compared to the pH of the soil after treatment (see Fig. 5). It can be observed that the pH throughout the soil remains more or less constant as the changes induced by polarity reversal are slower in the mass of the soil due to the higher buffering capacity of the soil; thus, the induced polarity change succeeded in maintaining the pH relatively constant as opposed to the solutions in the electrolyte chambers which react comparatively quite quickly to a reverse of polarity, hence the observed marked changes in pH in the electrolyte chambers. As reported earlier, the carbonic anhydrase hydrolyses CO_2_, forming a weak carbonic acid and lowering the overall pH of the soil compared to pH values noted in the electrolyte chambers (Supuran 2016); however, the pH in the soil was high enough throughout the test to prevent calcite dissolution, and also the pH value of the soil is seen to be on average about 8.00, which is the optimal pH for *B. licheniformis* CA enzymatic activity, according to the results shown in Fig. 3b. Thus, it can be concluded that pH conditions conducive to CaCO_3_ precipitation were maintained throughout the experiment.

**Fig. 6:**
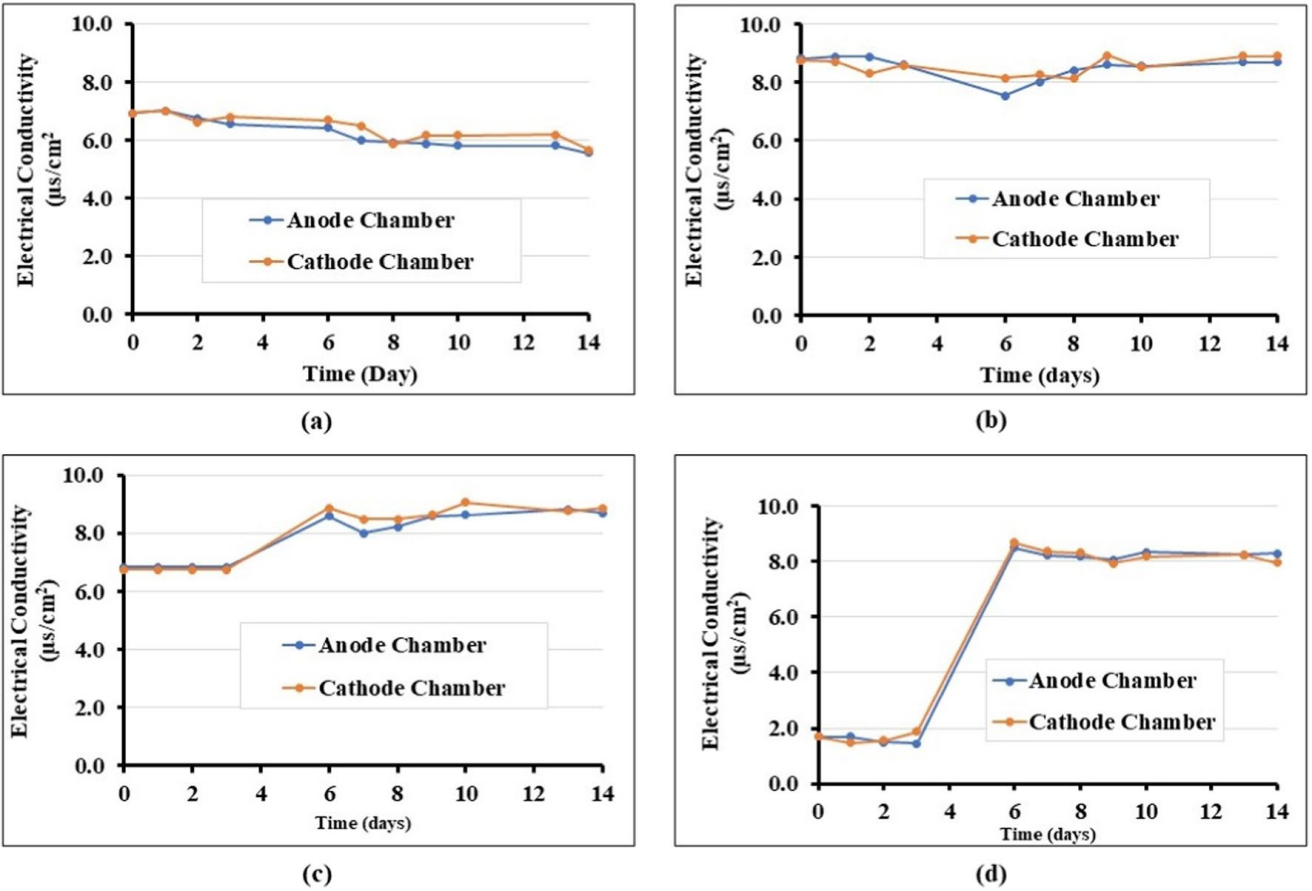
Electrical conductivity changes of the anode and cathode chambers during various treatment procedures: **a** nutrients only; **b** cementation solution only; **c** biostimulation; **d** bioaugmentation using *B. licheniformis*. Note: “Anode” and “Cathode” chambers refer to the electrolyte chamber function when injection started

The changes in electric conductivity (EC) of the different solution fluids at the anode and cathode chambers during the tests were measured to understand the ability of the reagents to transmit electrical currents. It is well known that the ability of a medium to conduct electricity is directly proportional to the concentration of conductive ions. The results of EC for all the cases are shown in Fig. 6a–d. The initial EC values for nutrients only and the cementation solution only were 6.93 µs/cm^2^ and 8.75 µs/cm^2^, respectively. For nutrients only, the EC decreased to about 5.66 µs/cm^2^ upon EC treatment, as mineral nutrient components of the solution consumed for microbial growth cause the EC to reduce steadily. A similar trend was observed in the case of treatment with cementation solution only, as the calcium ion was consumed by precipitation during the experiments (for treatment with cementation solution only, CaCO_3_ precipitation was observed both in the soil—see Fig. 9b—and the electrolyte compartments). However, the biostimulation and bioaugmentation cases show an initial low EC of 1.69 µs/cm^2^ and 6.75 µs/cm^2^, respectively; then, when cementation solution was added on day 3, the EC increased to 8.86 µs/cm^2^ and 8.87 µs/cm^2^. This can be attributed to the Ca^2+^, C_2_H_3_O_2_^−^, Na^+^, and HCO_3_^−^ from the components. At the end of the test, these values decreased to 8.29 µs/cm^2^ and 8.70 µs/cm^2^ in both electrolyte chambers, due to CaCO_3_ precipitation.

**Fig. 7:**
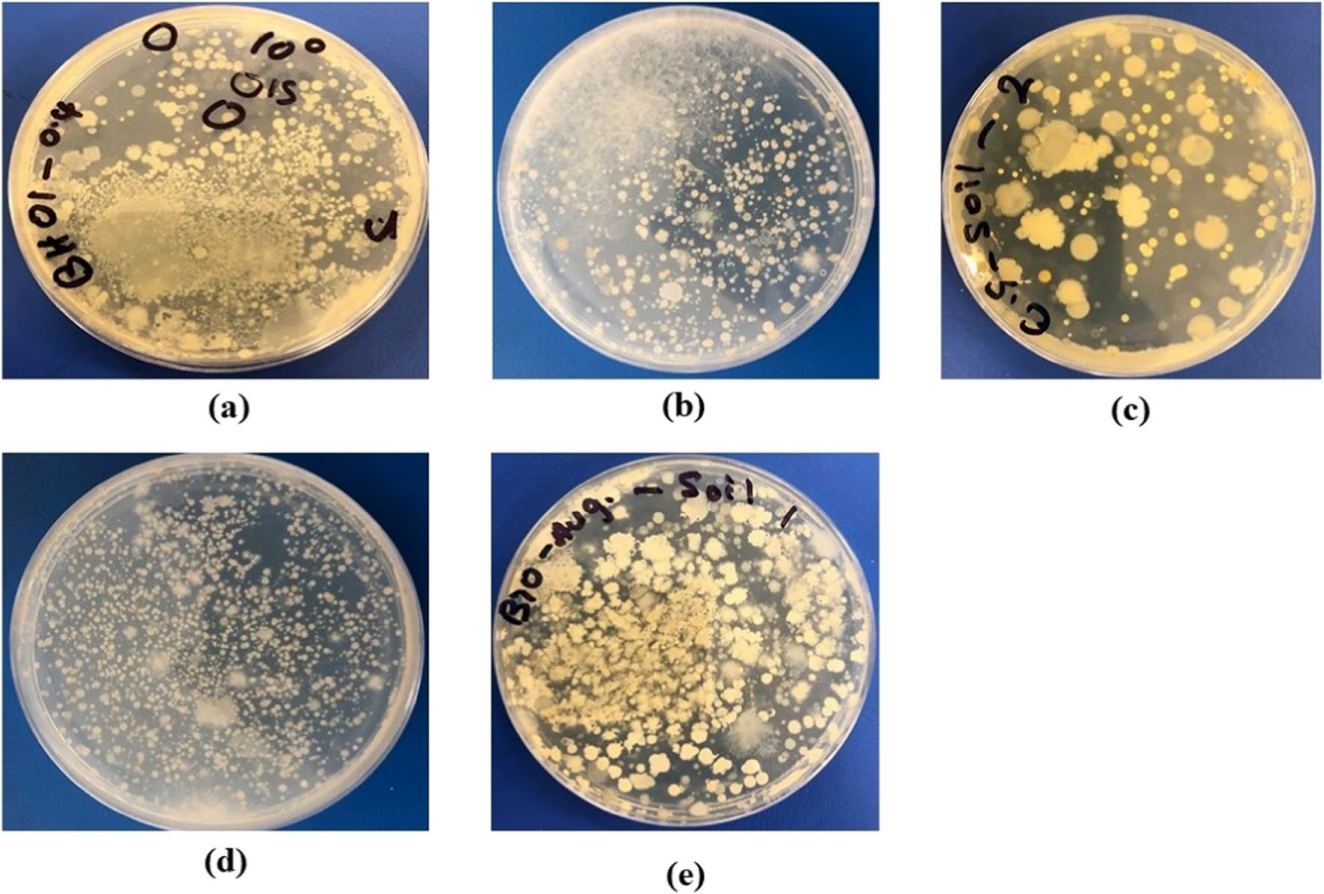
Microbial growth on agar plate of soil for **a** untreated soil; **b** nutrients only; **c** cementation solution only; **d** biostimulation; **e** bioaugmentation

A qualitative method of plating the soil before and after treatment (Fig. 7a–e) was carried out to (a) confirm that the electrical potential applied during EK biocementation did not alter the microbial activity of the soil and (b) to verify that the bacteria supplied during the bioaugmentation treatment entered the fine-grained soil (this has been debated in the biocementation literature, where the general belief is that due to the pore throat size of clays, bacteria cannot move or survive, see e.g., Mitchell and Santamarina 2005). Plating allowed us to assess whether bacteria had survived during the EK treatment and to make qualitative observations on the morphology of the colonies (see Fig. 7). Namely, as previously reported, inoculum during bioaugmentation could survive, grow quickly, and become the predominant microorganisms after inoculation. On the other hand, in the biostimulation treatment, the microbial community composition is more even and rich than in the augmented strategy as observed in this and previous studies (Chaudhary et al. 2021; Wu et al. 2019). During plating, bacteria were detected both before and after treatment, confirming that bacteria survived the EK process which is a valuable finding. Additionally, for the bioaugmentation case the plates showed predominantly one type of colony morphology, which was consistent with the morphology of *B. licheniformis*, previously observed by the authors. This implies that this was the injected single strain into the soil (*B. licheniformis*). This confirms that externally supplied bacteria reached the soil, an important finding. Conversely, in the biostimulation case, plates showed different strains as the stimulation solution encouraged the growth of different strains of bacteria in the soil. Note the varied colonies also in the cases of the untreated soil and nutrient-only treatment. This was expected, as the clay soil used in this study was not sterilised so that we do not affect the structure and physico-chemical characteristics of the clay soil. Namely, it has been widely demonstrated in the literature that autoclaving (the sterilisation method we usually adopt in our laboratory) affects the soil pH, decreases the cation-exchange capacity (CEC), and increases the dissolved organic carbon (DOC) and electrical conductivity (EC). Moreover, autoclaving increases available/exchangeable/extractable nutrients, e.g., N (NH_4_^+^ and NO_3_^−^), P, Mg, Mn, and Fe. Similarly, sterilisation through γ-irradiation would also affect the soil physicochemical characteristics and nutrient availability, although it is considered relatively less disruptive (Mahmood et al 2014). Identifying comparatively the microbial community of the strains through sequencing before and after treatment will be a subject of future work.

#### Undrained shear strength

**Fig. 8:**
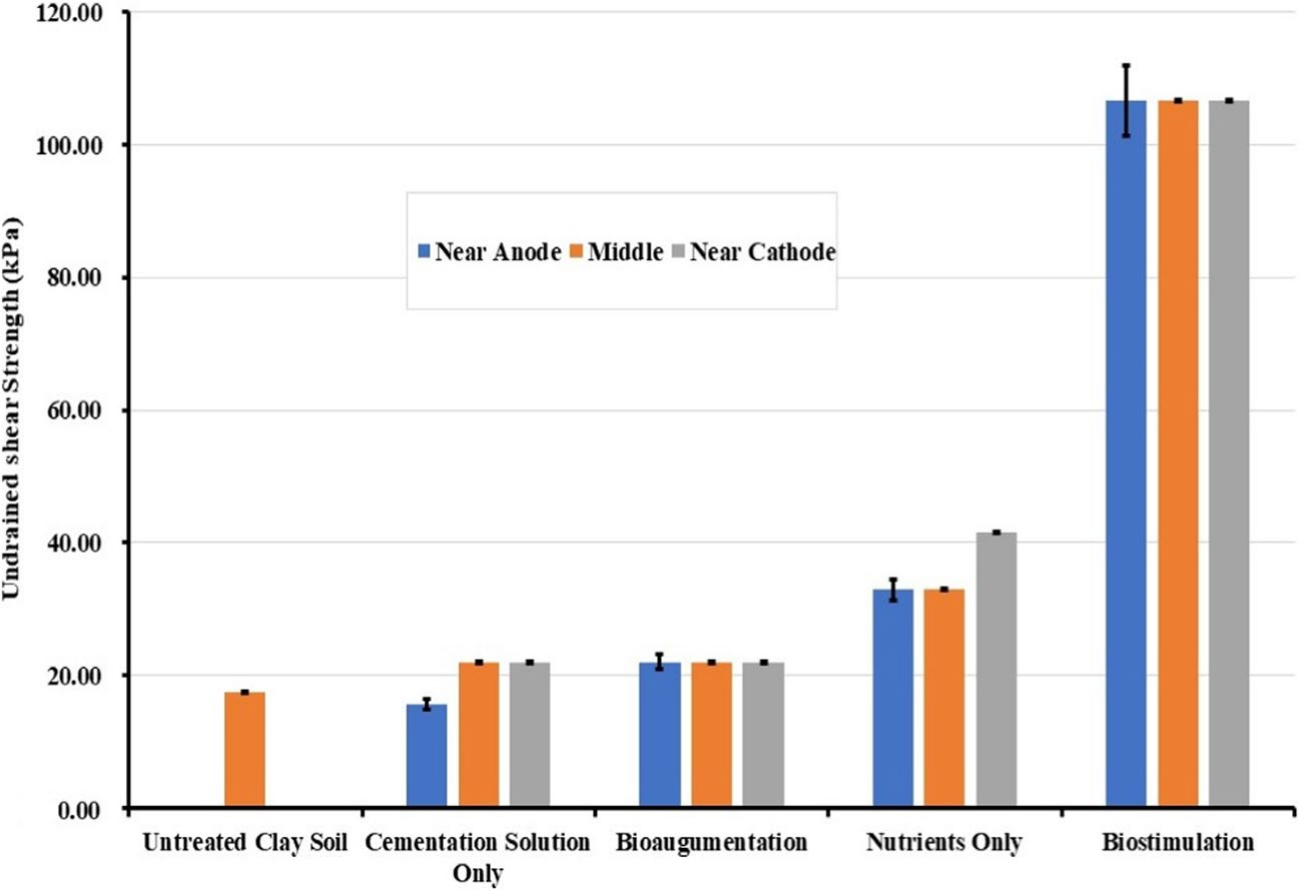
Undrained shear strength of untreated and treated clay soil. Note: “Anode” and “Cathode” refer to the electrolyte chamber function when injection started

Figure 8 shows the undrained shear strength of soil pre- and post-EK treatments, calculated according to Eq. 7 from cone penetration measurements. Biostimulation yielded an undrained shear strength *S*_u_ of 106.62 kPa, i.e., a value six times higher than the untreated soil whose *S*_u_ was 17.55 kPa. Interestingly, the specimens treated with nutrients only also had an increase in *S*_u_ of about 30 kPa, higher than those treated with cementation solution giving an *S*_u_ of about 20 kPa; this is difficult to explain as electroosmosis and electromigration would be expected to have similar effects for both cases and electromigration to be more pronounced in the cementing solution case due to Ca^2+^ ions. The bioaugmentation treatment did not increase the soil strength, although bacteria were confirmed to have entered the soil. Previous EK biocementation studies have shown that EK-bioaugmentation following the ureolytic biocementation pathway increased strength (Keykha et al. 2014; Safdar et al. 2021a, b); however, this was not observed here, possibly due to the low temperature at which the EK process was occurring whereas the optimal temperature for the bacteria used in this study was 37 °C. Due to the low temperature, the bacteria used in the bioaugmentation would not effectively produce enough carbonic anhydrase enzymes to promote calcium carbonate precipitation. On the other hand, biostimulation could have had a greater effect, as it relied on the whole population of native microorganisms, some of which also have sufficient CA enzyme activities at lower temperatures. Another key finding is the uniform distribution of strength increase using EK-biocementation across the soil sample (next to the electrodes and in the middle) and at different sample depths. EK, therefore, appears to be the answer in stabilising clay soils by biocementation, resolving the major issue of non-uniform treatments testified by all researchers using pressure injection of the treatments, and circumventing the requirement of developing high pressures in the soil during pressure injection, which would be unsuitable for fine-grained foundation soils of existing infrastructure.

#### Moisture content of the soil

**Fig. 9:**
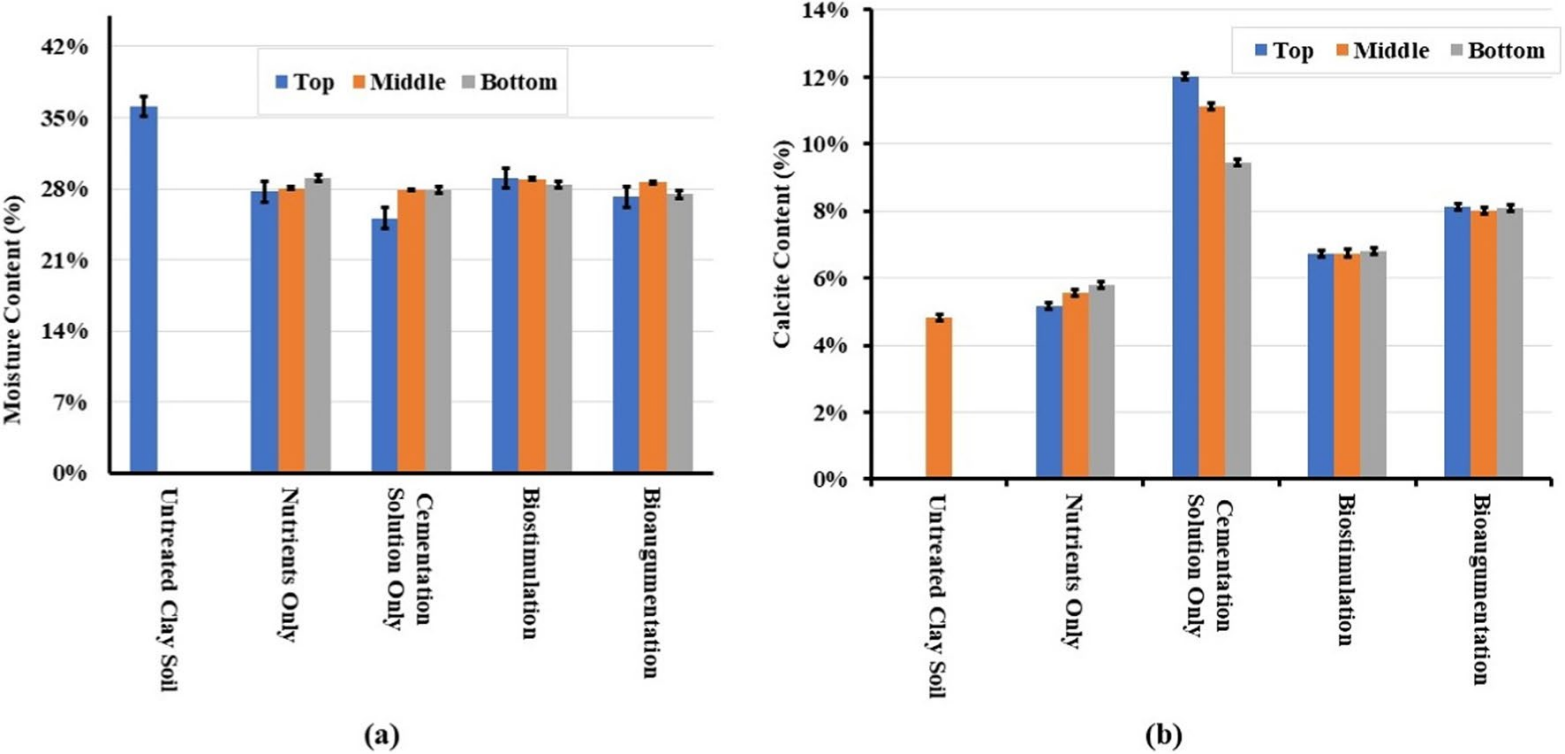
**a** Moisture content of the soil sample (top, middle, and bottom of the sample) before and after treatment. **b** CaCO_3_ content of the soil before and after treatment

The moisture content of the treated soil was measured for pre- and post-EK treatment on both ends of the box and the middle of the box at three different depths of the soil sample (Fig. 9). The top, middle, and bottom parts from each sampled part from the soil chamber in the EK set-up were analysed. The results showed a general reduction in moisture content for all the cases ranging between 7.2 and 9.2% (and consequently, no swelling of the soil was observed, as opposed to tests where treatments were implemented by pressure injection); this could be attributed due to electroosmosis from the anode towards the cathode, which was reversed every 24 h; hence, interstitial water was forced to move to and from both electrodes. No soil settlement was noted despite the small reduction in moisture content. On the other hand, the moisture content variations in the soil profiles from top, middle, and bottom of the soil samples taken from the EK chamber do not show any marked differences, as they ranged from 1.35 to 2.79% for all the cases, showing uniformity in the effect of the treatment on the moisture content. The reduction in moisture content could have affected the undrained strength of the soil to some extent. However, in the case of biostimulation, the undrained strength increase was considerably pronounced, and this would not be justified solely by the change in moisture content.

#### CaCO_3_ content of the soil

Figure 9b shows the CaCO_3_ content of the soil samples at the same locations as the water content and undrained shear strength, before and after the various treatments. The untreated clay soil contained already a considerable amount of calcium carbonate of about 5%, and an average of about 5.7% in the nutrient only case. This amount increased to 7% for biostimulation, 8% for bioaugmentation, and 12% for cementation solution only, whereas no significant changes were observed upon the injection of nutrients. The results were unexpected, as the measured CaCO_3_ contents were inconsistent with the strength increase results. Namely, biocementation by biostimulation should have logically resulted in the highest CaCO_3_ contents compared to the other treatments, as CaCO_3_ would be expected to bind the particles together, increasing the soil strength. It is possible however that the measured CaCO_3_ was not calcite in all instances; amorphous CaCO_3_ or unstable CaCO_3_ mineral forms like vaterite would not have resulted in a considerable strength increase. The increase in carbonate content in the cementation solution-only treatment could be attributed to the constituents of the solution itself, producing carbonate into the pores of the soil purely by chemical reaction; considerable amounts of precipitates were also observed on the electrodes inside the electrolyte compartments in the cementing solution treatment but also bioaugmentation case. However, it remains unclear why this purely chemical reaction would have produced higher CaCO_3_ contents than when bacteria and CA enzymes were present. It should be noted however that in the latter case, the produced bioprecipitates are of different quality and morphology than chemically (abiotically) produced precipitates and that the bacteria serve as nucleation sites for crystals to grow and bridge particles together, as attested in the literature, which could explain the better strengths during biostimulation. In the latter case (biostimulation), an increase of calcite content of about 1.3% as an average was observed compared to the nutrient-only solution; this was the only case where a significant UCS increase of approximately 90 kPa was observed compared to the untreated soil (or approximately 70-kPa increase compared to the nutrient only case), and we attributed this to calcite precipitation, as argued earlier. As the relationship between CaCO_3_ content and strength increase in microbially induced calcite precipitation (MICP) is soil dependent, it is difficult to compare with the literature, which deals mostly with sand biocementation. Indicatively however, we can mention that in Safdar et al. (2021b), who treated a soil with 50.8% organic content, CaCO_3_ contents of an average of 1.25% corresponded to an average UCS increase of 110 kPa, whereas Duraisamy (2016) performing sand biocementation by deep mixing recorded UCS between 120 and 200 kPa for CaCO_3_ contents between 0.8 and 1.33%, which are not very different to those recorded here for the biostimulation treatment. Finally, in Chittoori et al. (2021) who applied biocementation treatments to an expansive clay of 70% clay content, as well as to mixtures of this clay with sand, to obtain soils of 40% and 30% contents of clay respectively, UCS results were very variable depending on treatment protocol and CaCl_2_ content and did not always reflect measured CaCO_3_ contents. For example, UCS increases for the soil containing 40% clay (similarly to the soil in our study) of 47 kPa, 96 kPa, and 101 kPa were noted respectively for CaCO_3_ contents of 1.1%, 1.07%, and 0.97% for the lower CaCl_2_ content used or, for the higher CaCl_2_ content used, 25 and 32 kPa increases were noted for 0.82% and 0.68% of CaCO_3_, respectively. The highest UCS increase recorded for this soil for all treatment protocols (of 165 kPa) did correspond to the highest CaCO_3_ content achieved, i.e. 1.36%; however, generally it was much lower than UCS values reported for biocemented sands. The authors concluded that the precipitation of higher amounts of CaCO_3_ may not always result in improved engineering performance as other factors such as the type of CaCO_3_ formed as well as gradation, density, and other geotechnical characteristics also play an important role in UCS obtained during MICP.

#### SEM analysis

**Fig. 10:**
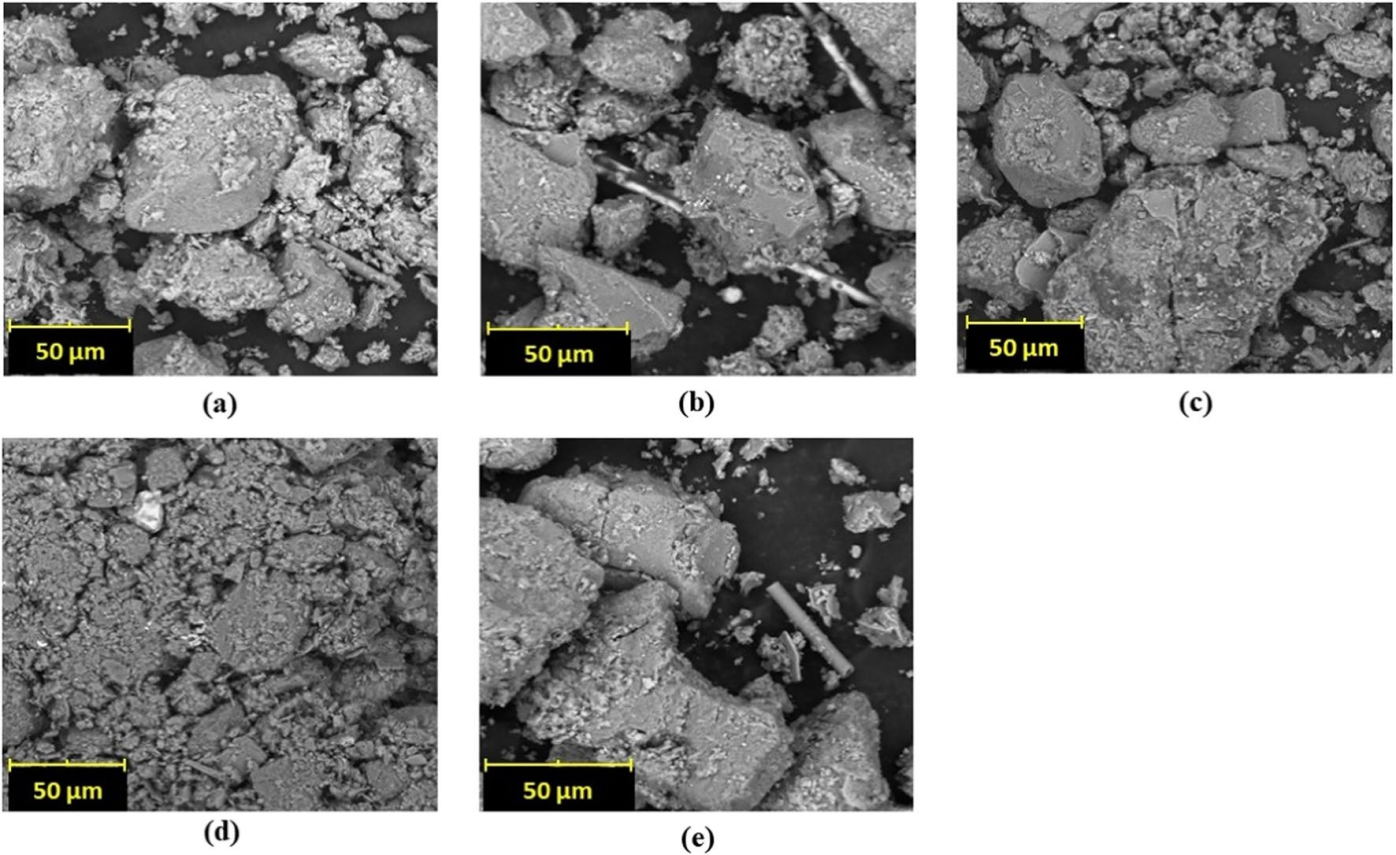
Scanning electron microscope micrograph for **a** untreated soil, **b** nutrients only, **c** cementation solution only, **d** biostimulation, and **e** bioaugmentation

The SEM was conducted on all treated specimens and the untreated soil to study the influence of stabilisation on microstructure and mineralogical characteristics. It can be observed that the untreated soil was morphologically similar to the control cases of nutrients only and cementation solution, as well as for the bioaugmentation (which is consistent with the limited shear strength gain). As shown in Fig. 10, the soil particles were plate-like in shape and parallel with each other. However, the biostimulation case (Fig. 10d) showed a different morphology of soil particles which were less parallel and reduced in size; they looked more aggregated and had a denser structure. This observation could be due to the action of the calcium in the calcium carbonate being formed during biocementation (calcium carbonate due to carbonation due to CA microbes in the soil) that could reduce the thickness of the diffuse double layer of clay through cation-exchange and flocculation-agglomeration reactions. This reaction could have promoted the increase in shear strength as described above. This observation (i.e. that there are no preferential orientations of the structure of treated clay) has also been reported elsewhere (Abdallah et al. 2023; Ogila 2021). Therefore, our study concluded that the flocculation and agglomeration between the soil particles contributed to the increase in the mechanical strength of the clay soil although it remains unclear why this was not also the case for the bioaugmentation treatment (Fig. 10e); this merits further study and experimentation with different bioaugmentation protocols.

## Conclusion

In this study, we investigated the feasibility of stabilising clay soil using EK via CA biocementation.

i. The results showed that this is possible, as the undrained shear strength of the treated soil became six times higher than that of the untreated soil when EK biostimulation was applied. For the same case, the SEM micrographs showed flocculation and agglomeration of soil particles, possibly due to calcium ions from calcium carbonate bridging the negatively charged clay particles.
ii. Biostimulation could be a preferred option for field implementation because the already present native microorganisms are well-suited to the subsurface environment and well-distributed. Conversely, the bioaugmentation treatment protocol needs to be revisited, as it did not lead to a considerable strength increase. However, it was confirmed that the pre-cultured bacteria entered the soil upon EK injection, which is an important finding as the general belief is that bacteria cannot be injected into clay soils due to the narrow pore throat sizes.
iii. Another important finding for practical purposes was the uniformity of the treated soil parameters across the soil sample and at different depths, which demonstrates that EK could be a most promising method for biocementation treatment implementation under existing infrastructure. EK can thus circumvent the widely documented issues of treatment non-uniformity if other common injection methods are used (such as pressure injection). Moreover, using EK, the development of high pressures which could be detrimental to the stability of existing earthworks, can be avoided.

Although these are early-stage findings and the research is ongoing, the prospects are exciting, as it was shown that it is possible to achieve the desired strength by biostimulation of native bacteria capturing CO_2_ while improving the soil strength, thus having the potential to contribute both to the resilience of existing infrastructure, as well as to climate change mitigation.

## Data Availability

The authors declare that the data supporting the findings of this study are available within the paper. Should any raw data files be needed in another format, they are available from the corresponding author upon reasonable request.
